# Extracorporeal circulatory systems in the interhospital transfer of critically ill patients: experience of a single institution

**DOI:** 10.4103/0256-4947.51792

**Published:** 2009

**Authors:** Assad Haneya, Alois Philipp, Maik Foltan, Thomas Mueller, Daniele Camboni, Leopold Rupprecht, Thomas Puehler, Stephan Hirt, Michael Hilker, Reinhard Kobuch, Christof Schmid, Matthias Arlt

**Affiliations:** a Department of Cardiothoracic Surgery, University Hospital Regensburg, Regensburg, Germany; b Department of Anesthesiology, University Hospital Regensburg, Regensburg, Germany; c Department of Internal Medicine II, University Hospital Regensburg, Regensburg, Germany

## Abstract

**BACKGROUND AND OBJECTIVES:**

Critically ill patients with acute circulatory failure cannot be moved to other institutions unless stabilized by mechanical support systems. Extracorporeal heart and lung assist systems are increasingly used as a bridge to end-organ recovery or transplantation, and as an ultimate rescue tool in cardiopulmonary resuscitation.

**PATIENTS AND METHODS:**

From July 2001 to April 2008, we had 38 requests for extracorporeal support for interhospital transfer carried out by the air medical service. Respiratory failure was present in 29 patients, who were provided with pumpless extracorporeal lung assist (PECLA) or veno-venous extracorporeal membrane oxygenation (ECMO). Cardiac failure dominated in 9 patients, who underwent implantation of extracorporeal life support (ECLS). Underlying diseases were acute respiratory distress syndrome in 15 patients, pneumonia in 7, prior lung transplant status in 4, cardiogenic shock in 7, and septic shock in 4.

**RESULTS:**

All assist systems were connected via peripheral vessels by the Seldinger technique. Transport was uneventful in all cases with no technical failures. On arrival at the specialized care hospital, two patients had leg ischemia and underwent relocation of the arterial cannula. After a mean (SD) support of 5.1 (3.0) days for PECLA, 3.5 (2.9) days for ECLS, and 7.3 (5.8) days for ECMO, 60%, 66%, and 66% of patients, respectively, could be successfully weaned from the systems. Discharge rates were 45% for PECLA, 44% for ECLS, and 56% for ECMO.

**CONCLUSION:**

Our experience proves that minimized extracorporeal assist devices allow safe assistance of patients with isolated or combined heart and lung failure in need of interhospital transfer. Critically ill patients get a chance to reach a center of maximum medical care.

Interhospital transfer of patients with acute cardiac failure (i.e., cardiogenic shock) to specialized medical centers is necessary for patient survival, especially in highly catecholamine-dependent and mechanically ventilated patients.^1^ Patients with severe respiratory failure (acute respiratory distress syndrome) as a result of generalized disease or severe trauma, including military personnel, are another group of patients frequently requiring transport to specialized care institutions. 2–4 The use of extracorporeal perfusion systems during transportation minimizes risk and avoids cardiovascular instability.^5^ There is little difference between transport within a clinical center or between locally separated institutions. The relevant logistic and security efforts are the same.^6^ This report reviews the technique and equipment required for interhospital extracorporeally assisted transport and evaluates patient outcome in our experience.

## PATIENTS AND METHODS

From July 2001 to April 2008, the Department of Cardiothoracic Surgery, University Hospital, Regensburg, Germany, had 38 requests for extracorporeal assistance in interhospital transfer missions carried out by the air medical service. The transport team included an anesthesiologist experienced in cardiopulmonary bypass, a perfusionist, a nurse or paramedic and a cardiac surgeon in cases of cardiogenic shock. Preparation time for the team was about 20 to 40 minutes after receiving a request. For air medical transfer, a rescue helicopter (Messerschmitt-Bölkow-Blohm BK-117) of the air rescue center in Regensburg was available. For ground transport, a special intensive care transport vehicle was used. After arrival of the transport team, a clinical assessment was performed to determine which assist device was most appropriate for the patient. The indication for extracorporeal lung- or heart- and lung-assistance is insufficient pulmonary gas exchange (hypoxemia/hypercapnia) during forced artificial ventilation. The appropriate system was assembled and primed, and the cannulas placed by the anesthesiologist and the perfusionist of the air medical service. Before initiation, 5000 units of heparin were given, except in patients with severe coagulopathy. Patient monitoring was the same as in the intensive care unit. Systemic anticoagulation was withheld for up to 24 hours after the start-up of extracorporeal assist as some patients had a high risk of bleeding (postopera-tive severe multiple trauma). Systemic anticoagulation was carried out via heparin perfusion pump. The effect of heparin was measured by the activated partial thromboplasin time. Patients without any particular risk of bleeding were given a dose of 50 mg to 100 mg acetylsalicylic acid (Apisol) every other day. In this case the aim was to assure the gas exchange capacity of the membrane oxygenator for the time of platelet aggregation inhibition.

In general, three systems are available for extracorporeal gas exchange. The underlying disease (isolated pulmonary failure or combined cardiopulmonary failure) defines the perfusion system required. The integral component of all systems is an extracorporeal module for gas exchange, i.e. an oxygenator. PECLA (pumpless extracorporeal lung assist) or interventional lung assist (iLA) is an extracorporeal gas exchange procedure designed by an interdisciplinary team at the Department of Cardiothoracic Surgery, University Hospital Regensburg in 1996.^7^ This arterio-venous bypass procedure uses the patient's blood pressure (the mean arterial pressure) as the driving force for the blood flow through an oxygenator. Accordingly, only patients with an adequate blood pressure and sufficient cardiac out-put are suitable candidates for this procedure. PECLA is primarily indicated in patients with inadequate elimination of CO_2_, such as in respiratory acidosis, as oxygen transfer is much less effective with this procedure.^8^ PECLA was developed to support pulmonary function in patients with severe respiratory insufficiency.

**Figure 1:**
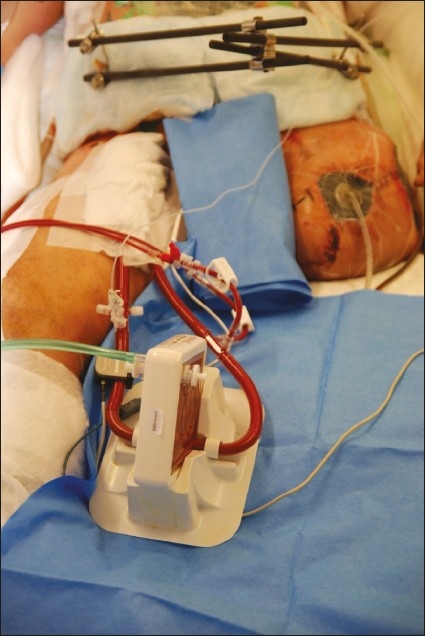
PECLA system with gas exchange membrane visible, along with ultrasound chip for measurement of blood flow.

The best indication is severe hypercapnia and moderate hypoxia.^9^ About 1-2 L/min are bypassed into the PECLA circuit. In our center, the PECLA membrane oxygenator (Novalung GmbH, 72379 Hechingen, Germany) is used on intensive care wards as well as for patient transport. The necessary priming volume is low (300 mL). Cannulas (NovaPort, Novalung, Hechingen, Germany) are placed via the Seldinger technique into the femoral artery (15-17 Fr) and femoral vein (17-19 Fr) (Figure 1). ECMO (extracorporeal membrane oxygenation) is a veno-venous extracorporeal bypass procedure used during severe global respiratory failure, such as acute respiratory distress syndrome, where cardiac pump function is not essentially affected. Via extracorporeal lung support sufficient gas exchange is created to treat critical hypoxemia and hypercapnia. The blood is drained from the inferior vena cava and diverted into the superior vena cava by a centrifugal pump (3-4 L/min). Lung protective ventilation may be added during extracorporeal gas exchange but is not essential. ECLS (extracorporeal life support) is a veno-arterial bypass procedure for emergency cardiopulmonary situations.

The indication for use is acute circulatory failure with or without pulmonary malfunction. Patients may suffer from primary heart disease (e.g. acute myocardial infarction) or from an unsuccessful intervention (failed PTCA or CABG).

**Figure 2:**
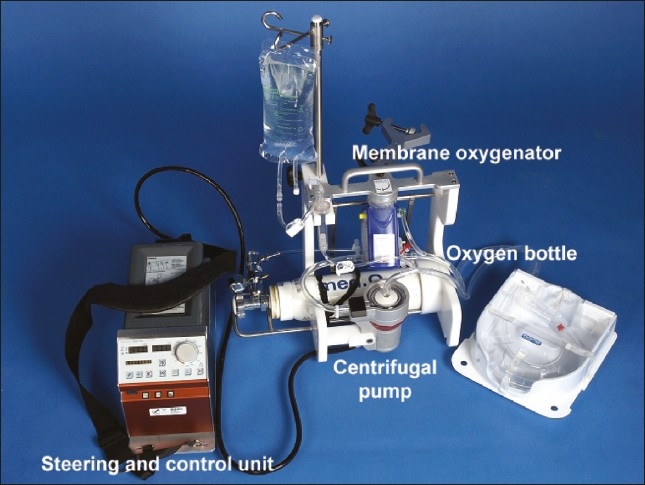
Emergency MECC system.

Transport of patients with severe cardiopulmonary failure is only possible with a mobile minimized extracorporeal support system (Figure 2). An emergency mobile minimized extracorporeal support system was developed in our institution, based on the MECC system from Maquet (Rotaflow, Maquet, Germany). It allows effective cardiorespiratory stabilization prior to and during transfer in an air or ground ambulance. The main components are a multifunctional holder with an oxygenator and a centrifugal pump, as well as a 2-liter oxygen bottle and a flow-regulator FM 41L 0-15 L/min (Dräger AG, Lübeck, Germany). The total weight of the equipped multifunctional holder is about 11 kg. The pump control is separate and provided with a carrying belt (16 kg). The complete system can be carried by one person, but can also be fastened on an ordinary stretcher of an ambulance vehicle. For combined life support and patient transport we use the “Emergency Life Support Set (ELS)” (Maquet Cardiopulmonary AG, 72145 Hirrlingen, Germany), which is heparin coated (Bioline Coating, Maquet Cardiopulmonary AG). The ELS set was modified as both Luer connectors were removed from the centrifugal pump after the priming (600 mL) to avoid air entrance in the negative pressure region of the extracorporeal circuit. All necessary components for percutaneous access (from covering cloths to cannula sets) were stored in a single box (60×40×35”). In patients with ECMO and ECLS systems, a 23 French femoral cannula (BFV 900-312, Sorin Group Munich, Germany) was used for venous drainage. In ECMO patients, the blood was returned via a cannula (15-17 Fr) into the internal jugular vein or the right subclavian vein. In patients with an ECLS, blood recirculation was into the common femoral artery. All cannulas were inserted percutaneously employing the Seldinger technique (Table 1).

**Table 1:** Blood flow and cannulation site for different techniques.

	Blood flow
iLA/PECLA	Femoral artery to femoral vein
ECLS	Femoral vein to femoral artery
ECMO	Femoral vein to jugular vein

ilA: interventional lung assist, PECLA: pumpless extracorporeal lung assist, ECLS: extracorporeal life support, ECMO: extracorporeal membrane oxygenation.

## RESULTS

A total of 38 patients in external hospitals were connected to the extracorporeal gas exchange systems prior to interhospital transfer (Table 2). Respiratory failure was present in 29 patients, who were provided with PECLA with or without ECMO (Table 3). Cardiac failure dominated in 9 cases, which underwent implantation of an ECLS. After local evaluation 20 patients were provided with PECLA systems, whereas 9 patients each were provided with ECLS and ECMO systems. Twenty-four of 38 patients were successfully weaned (63%) and 18 patients (47%) were discharged from the hospital (Table 4).

**Table 2:** Demographic data on the 38 interhospital transfer patients.

System	n	Mean (SD) age (years)	Gender (male/female)	Mean (SD) transport distance (km)	Transport (helicopter/ITV)
iLA/PECLA	20	35.9 (14.5)	17/3	227 (161)	14/6
ECLS	9	51.1 (8.9)	8/1	77 (23)	7/2
ECMO	9	40.9 (19.5)	5/4	105 (59)	7/2

ITV: intensive care transport vehicle, iLA: interventional lung assist, PECLA: pumpless extracorporeal lung assist, ECLS: extracorporeal life support, ECMO: extracorporeal membrane oxygenation.

**Table 3:** Diagnoses and extracorporeal systems used in 38 interhospital transfer patients.

Diagnoses	ILA/PECLA	ECLS	ECMO
ARDS post-trauma	8	-	3
ARDS	3	-	1
pneumonia	7	-	1
pre-lung transplantation	2	-	2
cardiogenic shock	-	6	1
Septic shock	-	3	1

**Total**	**20**	**9**	**9**

ARDS: acute respiratory distress syndrome, iLA: interventional lung assist, PECLA: pumpless extracorporeal lung assist, ECLS: extracorporeal life support, ECMO: extracorporeal membrane oxygenation.

**Table 4:** Overall mechanical support and patient outcome.

Group	n	Support (days)	Weaning (n)	Discharged from hospital (n)
iLA/PECLS	20	5.1 (3.0)	12	9
ECLS	9	3.5 (2.9)	6	4
ECMO	9	7.3 (5.8)	6	5

iLA: interventional lung assist, PECLA: pumpless extracorporeal lung assist, ECLS: extracorporeal life support, ECMO: extracorporeal membrane oxygenation.

Patients in the PECLA group had a mean (SD) PaO_2_/FiO_2_ ratio of 55 (5) mm Hg and a mean (SD) PaCO_2_ of 70 (10) mm Hg before device implantation. After referral to our institutions and further mechanical support with the PECLA system for 5.1 (3.0) days, 12 of 20 patients (60%) could be successfully weaned, while 8 patients (40%) died on the system. Finally, 9 of the 20 patients were discharged from the hospital with-out residual pulmonary impairment (45%).

Patients in the ECLS group were hemodynamically unstable and required a mean (SD) norepinephrine dosage of 7.6 (4.7) µg/kg/min to stabilize the circulation before placement of the veno-arterial bypass. Two hours after device insertion, the mean norepinephrine dosage could be reduced to 1.9 (1.1) µg/kg/min. During transport the pump flow was 3.0 (0.5) L/min. After continuous support for 3.5 (2.9) days, 6 of 9 patients were weaned. Four of 9 patients were discharged from the hospital (44%).

The two patients in the ECMO group had terminal lung failure. Accordingly, the intention of the device implantation was not only a safe transport to a primary care institution, but also a consecutive bridging to lung transplant, which was successful in both patients. Of the 7 remaining patients, 4 were weaned from the system after a total support of 7.3 (5.8) days, while 3 patients died during support. Eventually, 5 of the 9 patients were discharged from the hospital (56%).

During transport no malfunctions of the extracorporeal perfusion systems occurred, even though the mechanical demands (vibration) were considerable (e.g. during start and landing with a helicopter). During an interhospital transfer over the distance of 350 km an acute stop over became necessary on one occasion when the oxygen supply was insufficient. In another case, the gas exchange module of a PECLA system had to be exchanged after the recoiling hose assembly to the femoral vein had buckled for a short while and a partial thrombosis of the membrane oxygenator ensued. During in-hospital treatment, 2 patients with femorofemoral ECLS developed compartment syndrome in the respective lower extremity, which necessitated a relocation of the femoral artery cannula to the right subclavian artery. Another patient developed heparin-induced thrombocytopenia type II with thrombosis of the pump head on the sixth day of support. The pump head was changed and the anticoagulation changed to argatroban (Argatra, Mitsubishi Pharma Europe Ltd, London). The gas exchange capacity of the oxygenator was not affected so it was not necessary to exchange the oxygenator.

## DISCUSSION

The interhospital transfer of patients with the aid of extracorporeal perfusion systems is not a new procedure. 10–12 Foley and Bartlett had reported on more than 100 patients by 2002.^13^ However, considerable logistical, technical and human effort is required. The perfect operation of the extracorporeal circulation equipment without complications is important to successful interhospital transfer. Foley noted a complication rate of 17% associated with the system. Our approach in the development of transportable extracorporeal circulation systems was make the device as simple as possible including one module for gas exchange and one blood pump only. A new portable transport device for ECLS and ECMO was based on the MECC system of the Maquet company, which had been developed and routinely applied in our hospital in more than 1600 aortocoronary bypass operations.^14^ The PECLA system was identical to that used in the intensive care setting.

Air medical services using extracorporeal assistance pose a special challenge. The gas exchange capacity in the oxygenator decreases with the lowering of atmospheric pressure; in other words, if an oxygenator is used at high altitude, there is a reduced O_2_ transfer rate. Our flight altitude was always below 50 00 feet (1524 meters). As a result, the reduced atmospheric pressure had only a minimal effect on O_2_ transfer, with a mild desaturation of 3% to 4%. During a flight at 2300 meters (6900 feet), the oxygenator ventilated with an FIO_2_ of 1.0 would have the same capacity as at sea level with an FIO_2_ of 0.8. Forces of acceleration and forces of deceleration may affect the patient as well as the extracorporeal perfusion system during air transport, but we observed no blood volume shifting in the patient or perfusion system. We also had no problems with the venous return during take-off and landing, which may have been due to the use of helicopters instead of airplanes as helicopters mostly remain in a horizontal position.

Apart from the technical equipment, the qualifications and experience of the transport team plays a crucial role in the success of interhospital transfer. Experience in severe critical care patient management and percutaneous application of the cannulas is necessary as well as safe handling of the miniaturized perfusion system. In hospitals without vascular surgical stand-by, percutaneous arterial cannula placement carries considerable risk since small vessels may require surgical revision and anastomosis of a Dacron prothesis for cannula insertion. The placement of venous cannulas is less prone to complications.

In conclusion, our experience proves that miniaturized extracorporeal assist devices allow for the safe transport of critically ill patients with isolated or combined heart and lung failure.
